# Measuring pain intensity in older patients: a comparison of five scales

**DOI:** 10.1186/s12877-024-05127-6

**Published:** 2024-06-25

**Authors:** Sasikaan Nimmaanrat, Arpawan Thepsuwan, Suttipong Tipchatyotin, Mark P. Jensen

**Affiliations:** 1 Department of Anesthesiology, Faculty of Medicine, Prince of Songkla University, Hat Yai, Songkhla 90110 Thailand; 2 Department of Rehabilitation Medicine, Faculty of Medicine, Prince of Songkla University, Hat Yai, Songkhla Thailand; 3 Department of Rehabilitation Medicine, University of Washington, Seattle, WA USA

**Keywords:** Elderly, Geriatrics, Old, Older, Pain intensity, Pain scales, Pain tools

## Abstract

**Background and aims:**

Pain is common in older individuals. In order to understand and treat pain in this group, reliable and valid measures are needed. This study aimed to evaluate: (1) the validity, utility, incorrect response rates and preference rates of 5 pain rating scales in older individuals; and (2) the associations between age, education level, and cognitive function and both (a) incorrect response and (b) preference rates.

**Methods:**

Two hundred and one orthopedic clinic outpatients ≥ 65 years old were asked to rate their current pain, and least, average, and worst pain intensity in the past week using 5 scales: Verbal Numerical Rating Scale (VNRS), Faces Pain Scale - Revised (FPS-R), Verbal Rating Scale (VRS), Numerical Rating Scale (NRS), and Visual Analogue Scale (VAS). Participants were also asked to indicate scale preference. We computed the associations between each measure and a factor score representing the shared variance among the scales, the incorrect response and scale preference rates, and the associations between incorrect response and preference rates and age, education level, and cognitive function. The incorrect responses included being unable to respond, providing more than one response, responses outside a range, providing range answers rather than fixed answers, and responses indicating ‘least > average,’ ‘least > worst,’ and ‘average > worst’.

**Results:**

The findings support validity of all 5 scales in older individuals who are able to use all measures. The VNRS had the lowest (2%) and the VAS had the highest (6%) incorrect response rates. The NRS was the most (35%) and the VAS was the least (5%) preferred. Age was associated with the incorrect response rates of the VRS and VAS, such that older individuals were less likely to use these scales correctly. Education level was associated with the incorrect response rates of the FPS-R, NRS and VAS, such that those with less education were less likely to use these measures correctly. Cognitive function was not significantly associated with incorrect response rates. Age, education level and cognitive function were not significantly associated with scale preference.

**Conclusions:**

Although all five scales are valid, the VNRS evidences the best overall utility in this sample of older individuals with pain. The NRS or FPS-R would be fine alternatives if it is not practical or feasible to use the VNRS.

## Introduction

Pain intensity is the most common pain domain assessed in clinical and research settings [1]. It can be assessed using a variety of self-report scales, observational tools, and/or physiological measures. Among these, self-report scales are viewed as the gold standard, given the fact that pain is by definition a subjective experience [2]. Consistent with this idea, a position statement by the Australian and New Zealand Society for Geriatric Medicine, concluded that self-report should be viewed as the gold standard approach to pain assessment, and observational and behavioral measures should only be used for individuals unable to reliably indicate pain due to communication difficulties or severe cognitive deficits [3]. However, given that a number of self-report measures exist, each with its own strengths and weaknesses, there is not yet a consensus regarding which pain intensity measure(s) should be used for assessing pain intensity in older adults.

To help address this question, a number of studies have evaluated the psychometric properties of commonly used pain measures in older adults. The findings show that several assessment tools are valid for use in geriatric populations [4–7], although a number of important differences have been identified. For example, scale preference, which could potentially influence overall satisfaction and willingness to comply with an assessment procedure, have been shown to differ across different samples, with older individuals preferring the 0–10 Numerical Rating Scale (NRS) in some samples [4], the Verbal Rating Scale (VRS) in other samples [5], the Faces Pain Scales (FPS) in other samples [7], and the pain thermometer in still other samples [8]. The VAS has never been preferred over any other scale.

Research has also shown that level of cognitive impairment is associated with the ability of older individuals to use self-report measures, such that those with more cognitive impairment were shown to be less able to use pain measures correctly [9]. However, statistically significant associations between level of cognitive impairment and ability to use pain intensity scales are not always found [10–12]. One possible explanation for the discrepant findings may be related to between-study differences in how the self-report measures are presented to the respondents. The most common approach is to provide the respondent with a hard copy version of a measure, and ask them to respond using a pencil or pen. However, in clinical settings, patients are often asked to provide their rating verbally. This method of administration may make it easier to provide a correct response. If this were so, it could also be possible that a verbal version of pain measures may be more valid than or preferred over pencil-and-paper pain scales, especially among older individuals. To our knowledge, the evaluation of a verbal version of a commonly used measure has never been tested in a sample of older adults.

Given these considerations, the aims of the current study were to evaluate the validity and utility of 5 commonly used pain intensity scales in a sample of elderly patients with pain, including a verbal version as one of the scales to be evaluated, by (1) examining the scales’ associations with a factor score representing the variance shared among all five measures; (2) comparing the rates of incorrect responding as well as the type(s) of incorrect responses; (3) comparing the rates of scale preferences; and (4) examining the associations between age, education level, and cognitive function and both (a) incorrect response rate and (b) scale preference. Based on prior research, cited previously, we hypothesized that the findings would support the validity of all five scales as measures of pain intensity in a group of older patients who are able to use each scale correctly, as evidenced by strong associations with a factor score representing the shared variance of the five measures. With respect to scale utility, we hypothesized that the study participants would evidence higher rates of incorrect responding for the scales with more response options (i.e., the VAS and 0–10 NRS) than those with fewer response options (the FPS-R and 6-point VRS). With respect to the roles of age, education level, and cognitive function, we hypothesized that if significant effects emerged, the results would show that participants who were older, had lower education levels, and had greater cognitive dysfunction would evidence higher rates of incorrect responding than those who were younger, had more education, and had less cognitive dysfunction. We did not have any *a priori* hypotheses about the associations between age, education level, and cognitive function, and scale preference, as this has not yet been examined in prior research in older adults.

## Materials and methods

This study was approved by the Ethics Committee of Faculty of Medicine, Prince of Songkla University, Thailand (REC 63-050-8-1) (05/06/2020) and registered with ClinicalTrials.gov (ClinicalTrials.gov Identifier: NCT04555928) (21/09/2020) before data collection commenced. Anonymity of the data was maintained, in accordance with the Declaration of Helsinki.

### Participants

A non-probability convenience sample of 201 orthopedic clinic outpatients in southern Thailand were recruited into this cross-sectional study from 16 December 2020 to 29 March 2021. [Regarding the sample size calculation, Nunnally JC [13] suggested that the participant-to-item ratio should be at least 10:1. With this suggestion, we required at least 50 participants as we had 5 items (pain scales)]. Inclusion criteria were being ≥ 65 years old, endorsing having at least some pain in the past week, being able to speak and write in Thai, and not having motor deficits in the hands that would interfere with their ability to respond to paper-and-pencil questionnaires. Exclusion criteria were lack of fluency in Thai, having a neurological disorder or psychiatric illness that would interfere with participation, not being able to provide informed consent, and declining study participation.

### Procedures

Potential participants were approached by a research staff person while waiting for their clinic appointment. The study purpose and procedures were described to any individual who expressed interest in participation. Those who were found to be eligible were then asked to read and sign an informed consent form. They were then asked to provide demographic information and information about their pain via a paper-and-pencil questionnaire developed for this purpose. They were then administered the Thai Mental State Evaluation (Thai MSE, or TMSE) [14] to evaluate cognitive function. A great deal of evidence supports the MSE as a measure of cognitive function in many languages [15], including Thai [16]. The cut-off points for determining that someone has significant cognitive dysfunction (i.e., is at risk for having dementia) is ≤ 23 (out of a total score of 30).

Following the administration of the TMSE, the participants were provided instructions on how to use each assessment tool. These instructions were repeated for a maximum of 3 times if requested by the participant. The participants were then asked to rate their (1) current pain intensity as well as their (2) least pain intensity, (3) average pain intensity, and (4) worst pain intensity experienced during the last week, using Thai versions of five different scales, including a Verbal Numerical Rating Scale (VNRS), the Faces Pain Scale - Revised (FPS-R), a Verbal Rating Scale (VRS), written 0–10 Numerical Rating Scale (NRS), and Visual Analogue Scale (VAS; see a more detailed description of each scale in the next section). The four hard copy versions of the scales were presented on separate pages, so participants were not able to easily refer to their previous responses when responding to each scale. The measures were administered in random order (using a Latin square design). In the event that any participant was unable to use a measure or answered incorrectly to any scale, the administrator did not attempt to facilitate a correct response (other than to repeat the instructions up to 3 times, if requested by the participant, as noted previously). After the participants rated their pain intensity using each scale, they were asked to identify the scale they most preferred, or to indicate no preference if that was the case.

### Pain intensity measures

Participants were asked to rate their current pain intensity, as well as their least, worst, and average pain intensity in the past week, using the Verbal Numerical Rating Scale, the Faces Pain Scale-Revised, the Verbal Rating Scale, the Numerical Rating Scale, and the Visual Analogue Scale.

**Verbal Numerical Rating Scale (VNRS).** The VNRS asks the respondents to rate the intensity of their (current or recalled) pain intensity on a 0 to 10 scale, with 0 = “No pain” and 10 = “Worst pain imaginable.” [17]. In the current study, the instructions for and responses to the VNRS were provided and obtained verbally only; no written materials were used. The participants were asked to state a number from 0 to 10 that best represented the intensity of their pain.

**Faces Pain Scale - Revised (FPS-R).** The FPS-R presents the respondents with 6 drawings of facial expressions that represent different levels of pain intensity [18, 19]. Respondents are asked to select the expression that best represents their pain intensity. Each facial expression is associated with a number from 0 to 10 (0, 2, 4, 6, 8, or 10), and the FPS-R score is the number associated with the face selected. Although the FPS-R was originally designed for use in children, it has also been used in adult populations, including the elderly and individuals with low literacy.

**Verbal Rating Scale (VRS).** The VRS (sometimes also referred to as a Verbal Descriptive Scale [VDS] or categorical scale) consists of a list of adjectives or phrases that describe increasing levels of pain intensity. A commonly-used 6-point VRS includes the descriptors “No pain”, “Very mild pain,” “Mild pain,” “Moderate pain,” “Severe pain,” and “Very severe pain” [20]. Each word or phrase has a number associated with it (in this case, 0–5), and the respondent’s VRS score is the number associated with the word or phrase chosen.

**Numerical Rating Scale (NRS).** The NRS consists of numbers (often, integers from 0 to 10, which are used in the 11-point NRS) where 0 indicates “No pain” and the highest number (e.g. 10) indicates a severe level of pain, such as “Worst pain imaginable.” [21]. We used the 11-point NRS in the current study. The participants were asked to select the number that best represented their pain intensity, and the number selected represented their NRS score.

**Visual Analogue Scale (VAS).** The VAS is a line (usually, 10-cm long, like the one used in this study) with the left end labeled as “No pain” and the right end labeled an extreme level of pain, such as “Worst pain imaginable.” With the VAS, respondents are asked to make a mark anywhere on the line that represents their pain intensity at or somewhere between the 2 extremes. The measured length from the “No pain” end to the mark made by the participants in cm (e.g., somewhere between 0 and 10 for a 10 cm long line) represents their VAS pain intensity score.

### Validity, utility, and preference criteria

**Scale validity.** The criterion validity of the five scales was evaluated by examining the association (expressed as an eigenvalue) of each measure with a factor score representing the variance contained in all five measures [22, 23]. This is based on psychometric theory [24] which hypothesizes that the shared variance of a group of measures tends to factor out error variance associated with each individual item or scale. As a result, factor score best represents the “true” variance of the domain assessed by each of the measures.

**Scale utility.** The utility of each measure was evaluated by determining whether or not the participants responded to it correctly. Five possible incorrect responses were defined *a priori*.

1. If the participant was unable to respond to a scale after repeated explanations (maximum of 3 times), the response was classified as an “unable to respond” incorrect response.
2. If the participant provided 2 or more answers to the same measures (e.g., placed 2 or more marked on the VAS, selected 2 or more faces on the FPS-R, selected 2 or more numbers for the NRS, etc.), the response was classified as a “more than one response” incorrect response.
3. If the participant provided a response that was outside of the range of the response options (e.g., if they said “12” on the VNRS, or made a mark to the right of the extreme end on the VAS), the response was classified as an “outside the range” incorrect response.
4. If the participant provided a range of rather than a fixed answer (e.g., “it ranges from 3 to 5” when asked to indicate their pain intensity on the 0–10 NRS), the response was classified as “response range” incorrect response. Note that this incorrect response is not the same as a response indicating that the pain intensity level lies *between* two adjacent response options, which was viewed as a correct response (see below).
5. If the participant provided a least pain intensity rating that was greater than the average pain rating, a least pain intensity rating that was greater than the worst pain rating, or an average pain rating that was greater than the worst pain rating, the responses were classified as a “least > average,” “least > worst,” and “average > worst” incorrect response, respectively.

Any response that was consistent with the instructions (i.e., anything other than one of the five incorrect response types described above) was classified as a correct response. Note that respondents were allowed to provide a response that was between 2 response options (e.g., “6.5” on the NRS, or indicated that their pain intensity lied somewhere “between” 2 facial expressions). In such cases, the score would be that which was between the numbers associated with the 2 response options (e.g., “3” if they said that their pain intensity lied between the facial expressions that had scores of 2 and 4).

**Scale preference.** Each participant was asked to select the scale that he/she most preferred, or to indicate that they had no preferences, if this was the case.

### Statistical analyses

We first computed descriptive statistics for the demographic and pain history variables, as well as responses to the pain intensity scales (means and standard deviations for continuous variables, and number and percent for categorical variables) to describe the study sample and study measures. Next, to evaluate the relative validity of the five scales, we conducted a principal components analysis of the scale responses. We anticipated that a single factor representing pain intensity would emerge from this analysis, using a scree test. We planned to examine the loadings for each scale associated with the factor that emerged from the factor analysis, using these loadings as indications of each scale’s validity as a measure of pain intensity. To test the hypothesis regarding the differences in rates of incorrect responding, we compared the rates of incorrect responses across the five measures using a chi-square analysis. In the event that a significant omnibus effect for group differences emerged, we then planned to compare the rates between each pair of scales using chi-square analyses. Finally, in order to evaluate the associations between age, education level, and cognitive function on the one hand and incorrect response rates on the other, we conducted a series of 15 chi-square analyses, three for each scale. One examined the association between age group (< 75 years old and ≥ 75 years old) and incorrect response rate, the second the association between education level (lower vs. higher) with incorrect response rate, and the third the association between cognitive function (evidence for cognitive dysfunction vs. lack of evidence for cognitive dysfunction) and incorrect response rates. All data analyses were conducted using R program version 3.1.1 (Vienna, Austria).

## Results

### Participants and descriptive information about the study variables

Descriptive information about the study sample is presented in Table 1. As can be seen, 201 individuals were enrolled into the study. Almost three quarters were women (70%) and married (69%). The average age of the sample was 72 years (SD, 5). About half (51%) had a primary school education and about one-third had a bachelor’s degree or higher. Additional details about the study sample can be seen in Table 1.

**Table 1:** Demographic data (*N* = 201)

Variables		Mean ± SD			*N* (%)	
Female sex					137 (68%)	
Age (years)		72 ± 5				
Marital status						
Married					138 (69%)	
Never married					13 (6%)	
Separated/divorced/widowed					50 (25%)	
Highest level of education						
No formal education					1 (< 1%)	
Primary school					102 (51%)	
Junior high school					20 (10%)	
Senior high school					12 (6%)	
Vocational certificate					6 (3%)	
Bachelor degree or above					60 (30%)	
TMSE	Age					
	< 75 years (*N* = 143)		≥ 75 years (*N* = 58)	Total (*N* = 201)		P-value
Average TMSE score (median, IQR)	25 (23.50,26.00)		25 (23.20,25.90)	25 (23.50,26.00)		0.864
TMSE score (N, %)						0.813
: ≤ 23 : > 23	33 (23%) 110 (77%)		15 (26%) 43 (74%)	48 (24%) 153 (76%)		

TMSE: Thai Mental State Examination

Table 2 presents the sample means and standard deviations of the five scales evaluated in this paper.

**Table 2:** Means and standard deviations of the pain ratings using 5 measurement tools (*N* = 201)

Variables*	Mean ± SD
Current pain intensity	
VNRS	3.61 ± 2.67
FPS-R	3.29 ± 2.92
VRS	2.17 ± 1.31
NRS	3.89 ± 2.78
VAS	3.42 ± 2.70
Least pain intensity in the past week	
VNRS	3.26 ± 1.93
FPS-R	2.87 ± 2.34
VRS	1.96 ± 0.99
NRS	3.28 ± 2.05
VAS	3.07 ± 2.30
Average pain intensity in the past week	
VNRS	4.48 ± 2.13
FPS-R	4.18 ± 2.52
VRS	2.50 ± 1.01
NRS	4.65 ± 2.25
VAS	4.43 ± 2.30
Worst pain intensity in the past week	
VNRS	6.27 ± 2.48
FPS-R	5.93 ± 2.75
VRS	3.14 ± 1.10
NRS	5.99 ± 2.42
VAS	5.93 ± 2.66

FPS-R: Faces Pain Scale - Revised, NRS: Numerical Rating Scale, VAS: Visual Analogue Scale, VRS: Verbal Rating Scale, VNRS: Verbal Numerical Rating Score

*The range of each scale is as follows: VNRS 0–10, FPS-R with 6 faces depicting numbers from 0–10 (0, 2, 4, 6, 8 and 10), VRS with the scale representing 0 (no pain), 1 (very mild pain), 2 (mild pain), 3 (moderate pain), 4 (severe pain) and 5 (very severe pain), NRS 0–10 and VAS 0–10

The Pearson correlation coefficients between each pair of scales are presented in Table 3. As can be seen, each scale evidenced a strong association with all of the other scales for each pain intensity domain (i.e., current, least, average and worst pain) with *r*’s ranging from 0.58 to 0.89

## Validity of the five measures

As expected, the scree test for the principal components analyses strongly supported the conclusion that the five scales assessed a single over-arching domain for each of the four pain intensity domains, with the first eigenvalue ranging from 3.72 to 4.17 and the second ranging from 0.74 to 0.83 (Table 4). Furthermore, all five scales demonstrated strong loadings (all eigenvalues > 0.75) on the single component that emerged from each analysis (Table 4). The NRS had the highest loading on the components representing current (0.96), least (0.91) and average (0.91) pain, and the second highest loading on worst pain (0.92). The VNRS had the highest loading on worst pain (0.93) with the second highest loading on current (0.92), least (0.87) and average (0.87) pain.

**Table 3:** Inter-scale correlation coefficients

Scale Correlation		Pearson correlation coefficient (*r*)			
		Current pain	Least pain	Average pain	Worst pain
VAS	VNRS FPS-R VRS NRS	0.80*** 0.71*** 0.78*** 0.89***	0.69*** 0.65*** 0.65*** 0.75***	0.66*** 0.66*** 0.66*** 0.74***	0.79*** 0.61*** 0.75*** 0.80***
VRS	VNRS FPS-R NRS	0.79*** 0.72*** 0.85***	0.67*** 0.60*** 0.72***	0.72*** 0.61*** 0.73***	0.80*** 0.61*** 0.77***
NRS	VNRS FPS-R	0.88*** 0.77***	0.78*** 0.69***	0.79*** 0.65***	0.84*** 0.61***
FPS-R	VNRS	0.71***	0.62***	0.58***	0.65***

*** P-value < 0.001

FPS-R: Faces Pain Scale - Revised, NRS: Numerical Rating Scale, VAS: Visual Analogue Scale, VRS: Verbal Rating Scale, VNRS: Verbal Numerical Rating Score

**Table 4:** Component loadings from the principal components analyses of the 5 rating scales

Scale	Current pain	Least pain	Average pain	Worst pain
VNRS	0.92	0.87	0.87	0.93
FPS-R	0.85	0.82	0.80	0.78
VRS	0.91	0.84	0.86	0.89
NRS	0.96	0.91	0.91	0.92
VAS	0.92	0.87	0.86	0.90
First 2 eigenvalues	4.17, 0.83	3.73, 0.75	3.72, 0.74	3.90, 0.78

FPS-R: Faces Pain Scale - Revised, NRS: Numerical Rating Scale, VAS: Visual Analogue Scale, VRS: Verbal Rating Scale, VNRS: Verbal Numerical Rating Score

Table 5 presents the findings regarding the rates of incorrect responding as a function of measure and type of incorrect response. As can be seen, every participant provided a response to each scale, and very few participants provided a range of responses. Overwhelmingly, the most common incorrect response types were those related to rating the different intensity domains in ways that suggest either or both (1) a lack of understanding of the concepts of worst, least, and average pain, or (2) problems with the measures for being able to rate differences between these pain domains. For example, across the five scales (5 × 201 subjects = 1005 possible times), participants rated the least pain as being greater than average pain 102 times (10%), least pain as being greater than worst pain 24 times (2%), and average pain as being greater than worst pain 71 times (7%). The total number of incorrect responses was largest for the VAS.

**Table 5:** Rates of incorrect responding for the 5 rating scales

Scale	Types of incorrect response (*N*, %)						
	More than 1 response*	Least > average	Least > worst	Average > worst	Mark between 2 responses	Unable to respond	Total
VNRS	0 (0%)	16 (1.6%)	1 (0.1%)	5 (0.5%)	0 (0%)	0 (0%)	22 (2.2%)
FPS-R	0 (0%)	25 (2.5%)	3 (0.3%)	10 (1%)	1 (0.1%)	0 (0%)	39 (3.9%)
VRS	0 (0%)	16 (1.6%)	6 (0.6%)	12 (1.2%)	0 (0%)	0 (0%)	34 (3.4%)
NRS	1 (0.1%)	16 (1.6%)	5 (0.5%)	18 (1.8%)	0 (0%)	0 (0%)	40 (4%)
VAS	0 (0%)	29 (2.9%)	9 (0.9%)	26 (2.6%)	0 (0%)	0 (0%)	64 (6.4%)
Total	1 (0.1%)	102 (10%)	24 (2.4%)	71 (7%)	1 (0.1%)	0 (0%)	199 (20%)

FPS-R: Faces Pain Scale - Revised, NRS: Numerical Rating Scale, VAS: Visual Analogue Scale, VRS: Verbal Rating Scale, VNRS: Verbal Numerical Rating Score

*For the more than 1 response and unable to respond incorrect responses, the numbers and percentages reflect those from all four pain domains

### Incorrect responses as a function of age, education level, and cognitive function

Participants who were ≥ 75 years old and those with lower education level evidenced higher rates of incorrect responding. The age effect was statistically significant for the VRS (*P* = 0.047) and VAS (*P* = 0.049). The education level effect was statistically significant for the FPS-R (*P* = 0.02), NRS (*P* = 0.004) and VAS (*P* = 0.017). Although the rate of incorrect responses was larger for individuals with worse cognitive function than individual with better cognitive function for all of the scales except the FPS-R, the between-group difference did not reach statistical significance for any of the scales (Table 6).

**Table 6:** Associations between age, education level, and cognitive function and incorrect response rates (*N* = 201)

Variables	Number of participants with incorrect responses (*N*, %)				
	VNRS	FPS-*R*	VRS	NRS	VAS
Age					
: < 75 years (*N* = 143)	13 (9%)	28 (20%)	15 (10%)	25 (18%)	33 (23%)
: ≥ 75 years (*N* = 58)	8 (14%)	7 (12%)	13 (22%)	11 (19%)	22 (38%)
P-value	0.464	0.337	0.047*	0.964	0.049*
Education level^#^					
: Lower education (*N* = 141)	18 (13%)	30 (21%)	23 (16%)	33 (23%)	46 (33%)
: Higher education (*N* = 60)	3 (5%)	5 (8%)	5 (8%)	3 (5%)	9 (15%)
P-value	0.163	0.020*	0.203	0.004*	0.017*
TMSE score					
: ≤ 23 (*N* = 48)	3 (6%)	9 (19%)	5 (10%)	9 (19%)	10 (21%)
: > 23 (*N* = 153)	18 (12%)	26 (17%)	23 (15%)	27(18%)	45 (29%)
P-value	0.413	0.951	0.571	≥ 0.999	0.328

* P-value < 0.05

^#^ Lower education = less than bachelor degree, higher education = bachelor degree and above

FPS-R: Faces Pain Scale - Revised, NRS: Numerical Rating Scale, TMSE: Thai Mental State Examination, VAS: Visual Analogue Scale, VRS: Verbal Rating Scale, VNRS: Verbal Numerical Rating Score

### Scale preference as a function of age, education level, and cognitive function

With respect to scale preference, the majority of participants preferred the NRS (35%). The VNRS and FPS-R had the same preference rate (both 24%). The VAS was the least preferred (5%). Neither age, education level, nor cognitive function were significantly associated with scale preference rates (Table 7).

**Table 7:** Associations between age, education level and cognitive dysfunction and scale preference (*N* = 201)

Factors	Scales					
	VNRS	FPS-*R*	VRS	NRS	VAS	*P*-value
Total preference (N, %)	49 (24%)	48 (24%)	23 (12%)	71 (35%)	10 (5%)	< 0.001
Age						0.188
: < 75 years (*N* = 143)	32 (22%)	36 (25%)	20 (14%)	50 (35%)	5 (4%)	
: ≥ 75 years (*N* = 58)	17 (29%)	12 (21%)	3 (5%)	21 (36%)	5 (9%)	
Education level^#^						0.883
: Lower education (*N* = 141)	37 (26%)	34 (24%)	15 (11%)	48 (34%)	7 (5%)	
: Higher education (*N* = 60)	12 (20%)	14 (23%)	8 (13%)	23 (39%)	3 (5%)	
TMSE score						0.650
: ≤ 23 (*N* = 48)	11 (23%)	14 (29%)	4 (8%)	18 (38%)	1 (2%)	
: > 23 (*N* = 153)	38 (25%)	34 (22%)	19 (12%)	53 (35%)	9 (6%)	

^#^Higher education = bachelor degree and above

TMSE: Thai Mental State Examination

FPS-R: Faces Pain Scale - Revised, NRS: Numerical Rating Scale, TMSE: Thai Mental State Examination, VAS: Visual Analogue Scale, VRS: Verbal Rating Scale, VNRS: Verbal Numerical Rating Score

## Discussion

The study findings are generally consistent with those from others that have compared different pain intensity measures in older individuals, and adds new information regarding the validity, utility, and preferences for a verbal numerical rating scale measure. The findings have important implications for determining which measures to use in this population in clinical and research settings.

With respect to scale validity, the findings indicate that for those who are able to use the scales, each can be considered valid, as reflected by their strong associations with a general pain intensity factor created via principal components analysis. This finding is consistent with other studies comparing the relative validity of different combinations of these scales in both older individuals and adults [4, 7, 25]. The new finding from the current study is that this conclusion extends to a verbal numerical rating scale.

It was demonstrated that a predominant proportion of incorrect responses pertained to average pain. This could be attributed to challenges encountered in valuing scores for average pain than least and worse pain. Average pain may be a more abstract construct than least or worst. Rating average pain as higher than worst pain and rating average pain as lower than least pain were previously demonstrated as the 2 most common errors across 4 pain measurement scale (VAS, VRS, NRS and FPS-R) [22].

It was found that the VAS evidenced the highest rate of incorrect responses. This may be attributed to the VAS format, which includes a wide range of pain ratings and labels with words on both ends without numbers or intervals. Hence, participants may face challenges in accurately positioning their responses along the line. This finding that the VAS exhibited the highest rate of incorrect responses is also consistent with prior research in both elderly individuals and adults [5, 22, 26–29], as is the finding that the NRS had a relatively low incorrect response rate [22, 29]. The one finding that is markedly discrepant from all of these is one that examined the relative incorrect response rates of individuals from Nepal [23]. In this study, the NRS evidenced a markedly high incorrect response rate of 64%, which was greater than that found for the VAS (33%), VRS (24%) and FPS-R (18%) in the sample studied. Part of the reason for this discrepant finding is that in the study examining the measures in individuals from Nepal, the respondents who provided a rating that was between two adjacent response levels (e.g., 6.5 instead of 6 or 7 on the 0–10 NRS) were classified as making an incorrect response. Given that some people are able to discriminate as many as 22 levels between no pain and severe pain [30], a response the lies between two response options when 20 or fewer options are allowed may not, in fact, be an error. For this reason, such responses were not classified as being incorrect in the current study. If this category of incorrect response was not considered incorrect in the Pathak et al. [23] study, then the NRS’s rate of incorrect responses would drop to 28%, which would be lower than that found for the VAS (31%) in that study.

Overall, and especially for the incorrect response categories other than rating least > average, least > worst, and average > worst pain, the incorrect response rates were incredibly low for the scales evaluated here - lower than those observed in many other studies. This may be due to the procedures which involved providing detailed instructions to participants regarding how to use the measures, and giving participants up to three times to ask clarifying questions. This suggests that if obtaining complete data with minimal errors is critical, such instructions should be a component of any pain assessment procedures [31].

Regarding the person factors associated with incorrect response rates, we found that older individuals, individuals with less education, and individuals with more cognitive dysfunction generally had higher rates of incorrect responses than those who were younger, had more education, and who evidenced better levels of cognitive function. These associations were statistically significant for some of the scales for both age and education level. However, and inconsistent with the study hypotheses, these effects were not uniformly larger for the measures in more response options (e.g., the VAS, NRS, VNRS) than those with fewer response options (FPS-R, VRS). For example, while significant age effects emerged for the VAS (a scale with many response options) as hypothesized, they also emerged for the VRS, which is the scale with the fewest response options studied here. Similarly, while significant education effects emerged for the VAS and NRS, they also emerged for the FRS-R. The only measure whose incorrect response rates were not significantly associated with any person factor was the VNRS, perhaps in part because of the very low rates of incorrect responding for this measure overall. This finding suggests that there may be something about communicating verbally about one’s pain that helps to ensure a better ability to rate pain intensity with fewer errors. This possibility should be examined in future research, as it suggests a strategy for minimizing incorrect responding in samples who are at greater risk for not using pain intensity scales correctly.

Regarding scale preferences, our participants most preferred the NRS. The VNRS and FPS-R tied in second place with respect to preference. Similar preferences were found from the other study with the NRS as the most preferred tool followed by the FPS-R [22]. However, one study identified the FPS-R was the most preferred scale followed by the NRS in postoperative adults varying in ages, including elderly with mild cognitive impairment [7]. Similarly, another study found that both cognitively impaired and intact older adults preferred the FPS over other pain intensity scales [11]. However, one study showed that the VRS was preferred over other tools in senior citizens [32]. Overall, the NRS and FPS-R tend to be rated as the most preferred tools over other measures most often, with one sometimes preferred over the other.

This study has a number of limitations that should be considered when interpreting the results. First, some of the positive findings with respect to the VNRS over the other measures may be due to the participants having greater familiarity with this scale, as it is the measure we routinely use in the setting where the research was conducted. Second, the majority of the participants were women, and about one-third of them had a bachelor’s degree or above. For all of these reasons, research in additional samples of older individuals, including samples that may be less familiar with the VNRS, samples with more men, and samples that include individuals with less education, is needed to help determine the reliability and generalizability of the current findings. Also, research emphasizing the influence of current pain on recalled pain and vice versa, across pain scales should be conducted to explore symptom memory and retrospective pain [33–35].

## Summary and conclusions

Despite the study’s limitations, the findings replicate previous research and provide important new information regarding the psychometric properties of commonly used measures of pain intensity in older individuals. The validity of all of the measures was supported in those who are able to complete the scales without errors. In addition, the findings provide significant support for the NRS and FPS-R in the population, and provide new findings with respect to the VNRS, supporting in particular the utility of this scale over paper-and-pencil scales. The findings suggest that when possible, it may be most useful to assess pain intensity verbally using a VNRS. When resources, the setting, or the design of a research study make a VNRS impractical, the results suggest that a NRS or FPS-R would be the excellent alternatives.

## Data Availability

The data that support the findings of this study are available on request to the corresponding author, S.N.
